# The Relationship Between Self-Control and Self-Efficacy Among Patients With Substance Use Disorders: Resilience and Self-Esteem as Mediators

**DOI:** 10.3389/fpsyt.2019.00388

**Published:** 2019-06-12

**Authors:** Chunyu Yang, You Zhou, Qilong Cao, Mengfan Xia, Jing An

**Affiliations:** ^1^School of Economics and Management, Changzhou Institute of Technology, Changzhou, China; ^2^School of Social and Behavioral Sciences, Nanjing University, Nanjing, China; ^3^Graduate School of Humanities and Social Sciences, University of Melbourne, Melbourne, VIC, Australia; ^4^Business School, Changzhou University, Changzhou, China

**Keywords:** self-control, resilience, self-esteem, self-efficacy, patients with substance use disorders

## Abstract

**Background:** While substance use disorder is one of the overarching health and social issues that might seriously disrupt individuals’ self-control and self-efficacy, most previous studies have been conducted among university students or other groups, and little is known about how the underlying mechanisms between self-control and self-efficacy might impact patients with substance use disorders.

**Objectives:** The purpose of this study is to investigate how resilience and self-esteem mediate the relationships between self-control and self-efficacy among patients with substance use disorders.

**Methods:** We conducted a cross-sectional study of 298 patients with substance use disorder from Shifosi rehab in China. Diagnostic and Statistical Manual of Mental Disorders (5th Edition)-based diagnostic questionnaires were used to collect demographic information and assess addiction severity. The Dual-Modes of Self-Control Scale (DMSC-S) was implemented to measure self-control, while self-esteem was measured using the Self-esteem Scale (SES). The Connor-Davidson Resilience Scale (CD-RISC) was used to measure resilience, and self-efficacy was measured by the regulatory emotional self-efficacy scale (RESE).

**Results:** The correlations between all the dimensions and total scores on the self-control, resilience, self-esteem, and self-efficacy were significantly positive (p < 0.01), indicating that they could predict patients’ self-efficacy. Bootstrap testing indicated that resilience and self-esteem fully mediated the relationship between self-control and self-efficacy, relationships between self-control and self-esteem were partially mediated by resilience, and resilience partially mediated the relationship between self-esteem and self-efficacy. Finally, the multiple-group analysis indicated that the relationships among self-control, resilience, self-esteem, and self-efficiency did not differ with respect to gender.

**Conclusions:** The path from self-control through resilience and self-esteem and on to self-efficacy is significant among patients with substance use disorders, suggesting that increasing self-control, resilience, and self-esteem can improve self-efficacy among patients with substance use disorders.

## Introduction

Substance use disorder is among the overarching health and social issues that could alter both psychological states and neural mechanisms (1–3), and a wide range of studies has suggested that illicit drug use might disrupt individuals’ self-control and self-efficacy capabilities (4–7). Although extensive studies have examined the relationships between self-control and self-efficacy (8–11), it is still complicated to draw general and incontestable conclusions about the complex relationships between self-control and self-efficacy, and one primary reason for this is that self-control and self-efficacy are both susceptible to situational factors (12, 13). In addition, since most correlational studies of such relationships have been conducted among university students or other groups (8, 10, 14), and few have dealt with patients with substance use disorder, it seems worthwhile to evaluate potential relationships between self-control and self-efficacy among patients suffering from such disorders.

### Self-Control and Self-Efficacy

Self-control is now widely conceptualized as a self-initiated ability that enables individuals to resist inappropriate or self-destructive temptations to achieve long-term goals. Historically, the definition of self-control has been developed from the concept of “effortful control” to the concept of “pursuing enduringly valued goals” (15). People with higher levels of self-control are more inclined to delay personal gratification based on instant impulses and allocate more well-resource energy to their future goals. At the same time, self-control is susceptible to various situational factors, including substance use disorders (13), family cohesion (16), and peer norms (17), and self-control has also been found to be closely correlated with a wide range of behaviors like psychological well-being (18, 19), academic performance (20, 21), and pathology (22, 23).

Self-efficacy plays a crucial role in forming self-judgment about whether one could realize intended goals. Self-efficacy can be separated into two types: general self-efficacy and task-specific self-efficacy. According to Bandura (12), general efficacy refers to an individual’s ability to perform across various situations, while task-specific self-efficacy is related to an individual’s ability to perform in a specific situation (that study assessed general self-efficacy). Many studies have suggested that people with high self-efficacy are more likely to be confident in coping with and handling resource-demanding tasks (24–26). According to Bandura (12), self-efficacy develops through individuals’ interactions with their surroundings because they are increasingly familiar with their ability to overcome tough tasks. The more demanding a task is, the less self-efficacy individuals may have, and they may then be less likely to engage in the task (27). Some studies also suggest that self-efficacy is closely associated with physical and mental health (25, 28), academic performance (29), and employment skills (30).

A large proportion of studies insist that self-control is positively correlated with self-efficacy (9, 10), and Bandura (31) suggested that self-control also plays a significant role in promoting self-efficacy. One interpretation of this underlying mechanism is that, on one hand, people with high self-control tend to pay more attention to their intended goals, while on the other hand, individuals with higher self-control are more likely to possess stable self-efficacy with respect to future tasks because they have successfully overcome similar hurdles in the past. Conversely, some studies have revealed that under specific circumstances self-control is negatively associated with self-efficacy (8, 32). Ein-Gar and Steinhart (8) revealed that people with low self-control might show higher self-efficacy when confronted with distant-future tasks. They may procrastinate not because of a failure in self-control, but because they have experienced sufficiently high self-efficacy before the task deadline. However, there is no general and incontestable finding as to the relationship between self-control and self-efficacy because both traits are susceptible to situational factors (12, 13).

Substance dependence can be a remarkable situational factor not only because it might alter individuals’ psychological states, but also it may modify their neural mechanisms. Numerous studies have suggested that consuming illicit drugs would most likely disrupt individuals’ self-control and self-efficacy capabilities (4–7), and many studies have verified that illicit drugs often lead to structural changes within specific brain regions, such as the prefrontal cortex (2) that modulates capabilities of balancing self-interests and aligning actions with internal goals (33), and those capabilities are the major components of self-control (15). Given the occurrence of psychological and physical alterations, while there might be heterogeneity between patients with substance use disorder and other groups with respect to analyzing relationships between these two traits, less attention has been given to examining the potential association between self-control and self-efficacy among patients with substance use disorder. To fill in this gap, this study integrated resilience and self-esteem as mediators to further analyze underlying mechanisms between self-control and self-efficacy among patients with substance use disorders.

### Resilience and Self-Esteem as Mediators

One specific mediator is resilience, defined as the ability to adapt to stress and negative emotions (34). Although few researchers have focused on the role of resilience in mediating between self-control and self-efficacy, there are numerous studies committed to exploring associations between self-control and resilience (35, 36), and association between resilience and self-efficacy (37–39). The findings of those studies showed that patients with higher levels of self-control are more likely to exhibit greater resilience than those with lower self-control (35, 40). Self-control acts as a protective factor to reduce the possibilities of feeling ashamed by providing resistance to temptation by inappropriate impulsion. It has been proven that shameful feelings are negatively correlated with resilience (41, 42), and there are many studies suggesting that resilience may predict self-efficacy. According to Schwarzer and Warner (43), adolescents with higher resilience are more likely to feel confident and be more effective and efficient in completing tasks, especially in high-effort situations.

Self-esteem serves as another mediator between self-control and self-efficacy by reflecting the evaluation made by individuals regarding their own worth (44). Extant research indicates that people with higher self-control tend to exhibit higher levels of self-esteem than those with lower levels of self-control (22, 45). Self-control might also contribute to various types of positive outcomes that act as indicators of self-esteem, such as better academic grades (46), better psychological adjustment (22), and better interpersonal relationships (47). Many other studies have also shown that self-esteem demonstrates the potential for fostering self-efficacy (48, 49) by providing self-confidence, regarded as a crucial component of self-efficacy (50).

Still, other studies have also identified close correlation between resilience and self-esteem. For example, Benetti and Kambouropoulos (51) published a study suggesting that resilience exerts a positive impact on self-esteem *via* a positive affect. Based on the fact that there are numerous studies evaluating the correlational roles of resilience and self-esteem, it seemed favorable for this study to select resilience and self-esteem as mediators between self-control and self-efficacy.

### The Present Study

**Figure 1:**
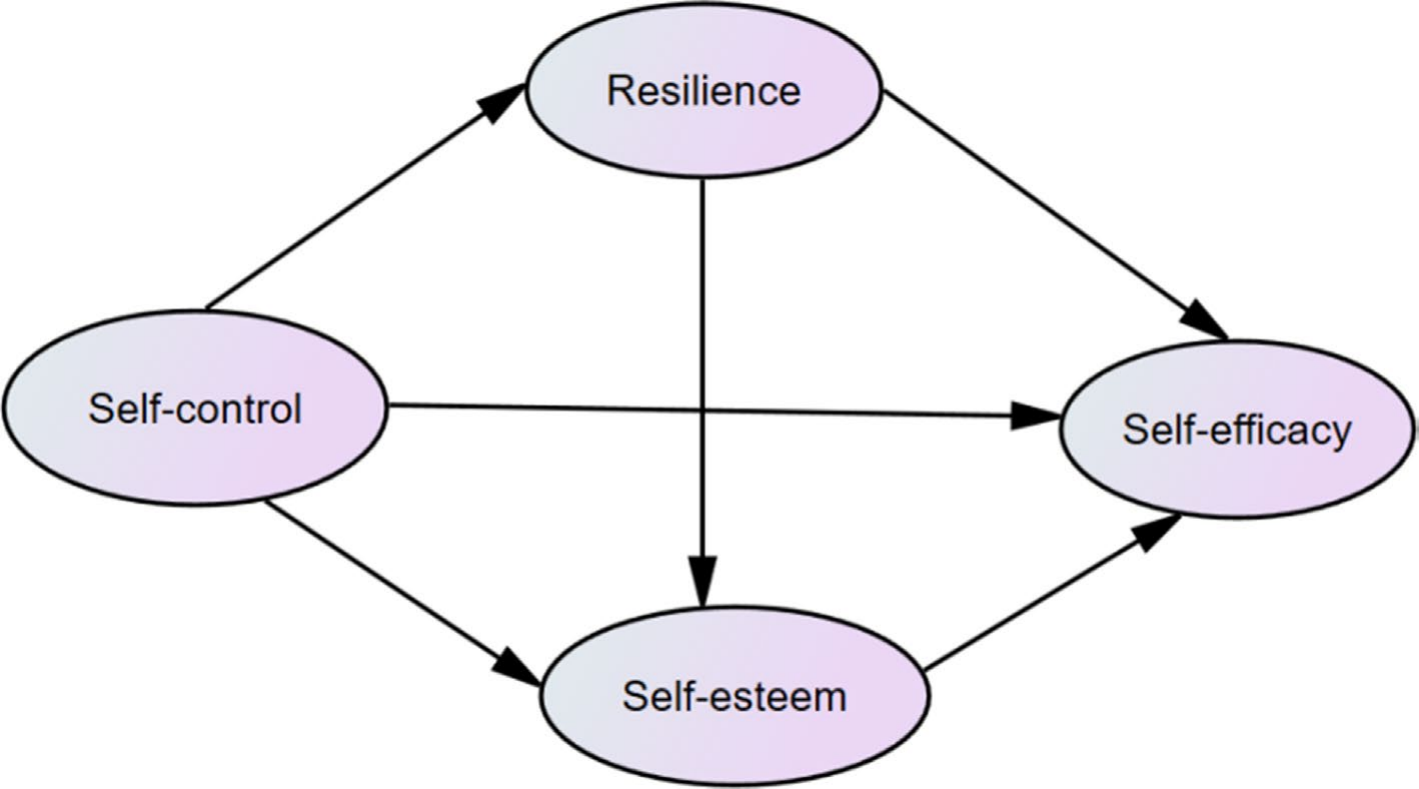
The hypothesized model concerning the relationship between self-control and self-efficiency: resilience and self-esteem as mediators.

There are numerous studies suggested that self-esteem and resilience have close links with self-control and self-efficacy (10, 22, 43, 45). Moreover, based on previous studies, a multiple-mediator model is more comprehensive than a single-mediator model with respect to conceptualizing the intermediary mechanisms (52, 53). In this study, we hypothesized resilience and self-esteem as mediators in the relationships between self-control and self-efficacy among patients with substance use disorder. Specifically, substance dependents with high self-control might ultimately experience higher levels of resilience and self-esteem to promote the functioning of self-efficacy. The detailed hypothesized model is presented in **Figure 1**.

## Method

### Participants and Procedure

The study was conducted at Shifosi Rehabilitation Center, a compulsory detoxification center, and involved 298 Chinese participants who had experienced substance use disorders. The Ethics Committee of Nanjing Medical University approved the study to ensure that it fully considered human rights, ethics, and safety throughout the procedures, and all participants signed informed-consent documents prior to the initiation of the assessment. To ensure confidentiality of the whole study, participants were separately placed in a separate conference room while completing the questionnaires in about 30 min.

The study dealt with seven socio-demographic characteristics of the participants: age, gender, education level, work status, years of use, substance classification, and DSM-5 addiction severity criteria. Inclusion criteria included the following: were of age of 18 years or more, exhibited normal and stable cognitive states, had been diagnosed with substance use disorders within the last 12 months, had normal vision and color perception, were right-handed, and had voluntarily agreed to participant in the study. Exclusion criteria included the following: a history of serious heart, liver, or kidney illnesses, cognitive disabilities, or psychiatric impairment caused by functional factors (e.g., physical illness, dysfunctions of neuroactive substance). The collective socio-demographic characteristics of the 298 participants are shown in **Table 1**.

**Table 1:** Sample characteristics.

Sample characteristics		Total (N = 298)		Male		Female	
		M	SD	n	%	n	%
Age	Male (18–64)	37.5	9.5	–	–	–	–
	Female (19–55)	35.2	8.2	–	–	–	–
		**n**	**%**				
Gender	1. Male	210	70.5	–	–	–	–
	2. Female	88	29.5	–	–	–	–
Education level: (n = 293)	1. Elementary school and below	49	16.4	35	16.7	14	15.9
	2. Middle school	125	41.9	87	41.4	38	43.2
	3. High school	88	29.5	61	29.0	26	29.5
	4. College	29	9.7	20	9.5	9	10.2
	5. Above college	2	0.7	2	1.0	0	0.0
Work status: (n = 289)	1. Unemployment	136	45.6	88	41.9	48	54.5
	2. Employment	134	45.0	108	51.4	25	28.4
	3. Others	19	6.4	11	5.2	8	9.1
Years of substance use: (n = 271)	<5 years	85	28.5	66	31.4	19	21.6
	6–10 years	94	31.5	62	29.5	32	36.4
	>10 years	91	30.5	57	27.1	34	38.6
Substance Classification	1. Heroin	82	27.5	60	28.6	22	25.0
	2. Methamphetamine	253	84.9	172	81.9	81	92.0
	3. Ketamine	53	17.8	30	14.3	23	26.1
	4. MDMA (ecstasy)	29	9.7	18	8.6	11	12.5
	5. Marihuana	33	11.1	19	9.0	14	15.9
	6. Others	8	2.7	6	2.9	2	2.3
Addiction severity	Mild	2	0.67	2	1.0	0	0.0
	Moderate	5	1.68	4	1.9	1	1.1
	Severe	291	97.65	204	97.1	87	98.9

The substance classes examined in the present study were matched with the drug classification of DSM-5 (54). Since some of the participants were polysubstance users (using more than one illicit substance), the sum of the prevalence with respect to drug classes exceeded 100%. As shown in **Table 1**, there were six drug classes: heroin (27.5%), methamphetamine (84.9%), ketamine (17.8%), methyenedioxymethamphetamine (MDMA)/ecstasy (9.7%), marihuana (11.1%), and others (2.7%).

The DSM-5 lists the following 11 symptoms of substance use disorders for assessing addiction severity: hazardous use, craving, withdrawal, tolerance, prolonged use of substantial amounts, collapse of relational and social relationships, withdrawal from social and occupational activities, use-related physical/psychological issues, substantial time spent using, repeated attempts to quit/control use, and social/interpersonal issues related to use (54). In DSM-5, addiction severity is measured by a criteria count: mild (from 2 to 3 criteria), moderate (from 4 to 5 criteria), and severe (from 6 to 11 criteria). The assessments were conducted using a diagnostic questionnaire that merged 11 DSM-5 criteria, and 291 of the participants (97.65%) were designated with the severest level of substance use disorders, while only 5 participants (1.68%) and 2 (0.67%), respectively, of the participants, were designated with moderate level and mild level disorders. The vast majority of patients were diagnosed with severe level of addiction because they were being treated in a mandatory drug treatment center that mainly tends to hospitalize patients with chronic substance use disorders.

### Measures

The study used a diagnostic questionnaire to acquire demographic information and assess the participants’ addiction severity levels. In the questionnaire, the participants self-reported demographic information such as gender, age, education level, and work status. The diagnostic questionnaire included three parts related to assessing addiction severity: substance use history, substance use behavior, and consequences and intervention history related to substance use. The diagnosis was based on 11 DSM-5 criteria. Finally, addiction severity was measured by counting the number of criteria matched in the questionnaires, resulting in a Cronbach’s α coefﬁcient of 0.70.

The Dual-Modes of Self-Control Scale (DMSC-S) was administered to assess participants’ levels of self-control. The scale consists of 21 items with responses on a ﬁve-point Likert scale from 1 = “not at all true” to 5 = “very true” (55). The DMSC-S is assessed using the impulse system and control system subscales. The impulse system subscale includes three factors: impulsive, easy distraction, and delay gratification, and the control system subscale includes two factors: problem-solving and future time view. The higher the score on the impulse system subscale, the stronger the factors of impulsiveness, distraction, and delay gratification, and the weaker the self-control ability. The higher the score in the control system subscale, the more likely the problem is solved satisfactorily, the stronger the future time view, and the stronger the self-control. The Cronbach’s α coefﬁcient in our study was 0.901.

The Connor-Davidson Resilience Scale (CD-RISC25) (56) was administered to assess participant resilience. The CD-RISC consists of 25 items with responses given using a ﬁve-point Likert scale from 0 = “not true at all” to 4 = “true nearly all of the time” (57). The total scores ranged from 0 to 100. The scale consists of three factors, viz., toughness, strength, and optimism, and the higher the score, the higher the resilience and the easier for an individual to recover when a stressful time is experienced. The Cronbach’s α coefﬁcient in our study was 0.939.

To assess participant self-esteem, we administered the Rosenberg Self-Esteem Scale (RSES) (58), designed to assess an individual’s overall perception of self-worth and self-acceptance. The RSES consists of 10 items, scored at four levels, with a total score of 10–40 points (59). The higher the score, the higher the degree of self-esteem. The scale includes items such as, “I feel that I am a valuable person, at least on the same level as others” and “I feel that I have many good qualities.” The Cronbach’s α coefﬁcient in our study was 0.711.

The Chinese version of the Regulatory Emotional Self-Efficacy Scale (RESS) was used to assess each participant’s evaluation of their ability to manage their emotions (60). This scale has two dimensions: perceived self-efficacy in expressing positive emotion and perceived self-efficacy in managing negative emotion (61). RESE consists of 17 items (e.g., “When the happy things happen, I will express my pleasure”), with responses using a five-point score ranging from 1 (completely disagree) to 5 (completely agree). The Cronbach’s α coefﬁcient in our study was 0.895.

### Data Analysis

We used initial correlational analysis to examine the relationships between self-control, resilience, self-esteem, and self-efficacy. Descriptive statistics and means and standard deviations (SD) were tested *via* IBM SPSS Statistics version 22.

In accordance with Anderson and Gerbing (62), we performed a two-step procedure to analyze mediation effects. We first used a measurement model that contained four potential variables: self-control, resilience, self-esteem, and self-efficacy, to test whether each latent variable could be well-represented by its indicators. We next determined whether the results from the measurement model were satisfactory; the structural model could be tested using maximum likelihood (ML) estimation in the AMOS 24.0 program. To control inflation of measurement errors generated by multiple items for the latent variable, we created several parcels using a random assignment method (63), and specially created three-item parcels for resilience and self-esteem, two-item parcels for self-control, and five-item parcels for self-efficacy.

To assess the adequacy of model fit, we used the following eight goodness-of-fit indices (64, 65): 1) chi-square statistics between 1 and 3; 2) a standardized root mean square residual (SRMR) of 0.06 or less; 3) a root-mean-square error of approximation (RMSEA) of 0.08 or less; 4) a goodness-of-fit index (GFI) of 0.90 or higher; 5) a Tucker–Lewis Index (TLI) of 0.90 or higher; 6) a comparative fit index (CFI) of 0.90 or higher; 7) Akaike information criterion (AIC); and 8) an expected cross-validation index (ECVI). We also used AIC and ECVI to compare two or more models, with a smaller value of AIC representing the better fit to the hypothesized model (66) and a smaller value of ECVI indicating a more significant potential for replication (67).

## Results

### Preliminary Analyses

The descriptive statistics including mean, SD, alpha, reliability estimates (Cronbach’s alpha coefficients), and correlations for all the study variables and clinical variables are shown in **Table 2**. They showed that, with respect to the clinical variables, there were significant negative correlations of age with gender, education level, and self-esteem, with gender on work status, and with work status on addiction severity, while there were significant positive correlations of age with years of addiction, with gender on addiction severity, self-control, and self-esteem, with education level on work status and self-efficiency, and with years of addiction on addiction severity. Moreover, all correlations among self-control, resilience, self-esteem, and self-efficiency were proven to be statistically significant (p < 0.01).

**Table 2:** Means, standard deviations (SD), Alpha, reliabilities, and intercorrelations among study variables.

Measure	Mean	SD	α	1	2	3	4	5	6	7	8	9	10
Age	36.8	9.17	–	1									
Gender^a^	–	–	–	−0.116*	1								
Education level	–	–	–	−0.135*	−0.005	1							
Work status	–	–	–	−0.026	−0.153**	0.122**	1						
Years of addict	10.3	7.71	–	0.594**	0.087	−0.085	−0.110	1					
Addiction severity	9.71	1.61	0.70	−0.048	0.220**	0.056	−0.209**	0.282**	1				
Self-control	65.87	8.04	0.901	0.022	0.149**	0.061	0.109	−0.021	0.042	1			
Resilience	82.27	17.62	0.939	0.020	0.059	0.034	0.047	−0.037	0.079	0.160**	1		
Self-esteem	25.79	2.74	0.711	−0.130*	0.156**	0.032	0.068	0.037	−0.104	0.292**	0.184**	1	
Self-efficacy	56.58	11.75	0.895	0.018	0.058	0.129*	0.035	0.038	0.054	0.171**	0.481**	0.231**	1

α = Cronbach’s alpha.

*Correlation is significant at the 0.05 level (two-tailed).

**Correlation is significant at the 0.01 level (two-tailed).

a Gender is coded 1 = male, 2 = female.

### Measurement Model

The measurement model included 4 latent factors: self-control, resilience, self-esteem, and self-efficacy, and 13 observed variables. Although the initial estimate was unsatisfactory, a revised model reflected satisfactory data: (χ^2^ = 156.67, df = 54, χ^2^/df = 2.901, p < 0.001; SRMR = 0.0495; RMSEA = 0.080; GFI = 0.927; TLI = 0.922; CFI = 0.946, and ACI = 256.667 ECVI = 0.864, CFA). All latent variable factor loadings were reliable (p < 0.01), and fitting results show that all latent variables could be well represented by the respective indicators.

### Structural Model

Without mediators, the direct path from self-control (the predictor) to self-efficacy (the criterion, r = 0.171, p < 0.01) was significant. We first built a fully mediated model (Model 1) containing two mediator variables (resilience and self-esteem) without a direct path from self-control to self-efficacy. While the initial results were unsatisfactory, a revised model produced satisfactory results [NC (χ2/df) = 2.751, RMSEA = 0.077, SRMR = 0.0561, GFI = 0.924, TLI = 0.912, CFI = 0.931, and AIC = 250.070], and all standardized path coefficients were significant (**Table 2**). Next, based on results from Model 1, a partially mediated model (Model 2) was tested by adding a direct path from self-control to self-efficacy, producing satisfactory test results: NC (χ2/df) = 2.780, RMSEA = 0.077, SRMR = 0.0563, GFI = 0.925, TLI = 0.911, CFI = 0.937, and Akaike information criterion (AIC) = 250.882, although the standardized path coefficient from self-control to self-efficacy in Model 2 was not significant. With respect to goodness-of-fit indices, while there was no noticeable difference between Model 1 and Model 2, there was one standardized path coefficient that was not significant in Model 2, so Model 1 was found to be better than Model 2. Next, in order to test the distal mediation effect, based on Model 1, a path from resilience to self-esteem (Model 3) was added to the model, with test results showing that, with respect to goodness-of-fit indices, while there was little difference between Model 1 and Model 3, the AIC and ECVI indices in Model 3 were smaller than for Model 1, indicating that Model 3 was better than Model 1.

**Figure 2:**
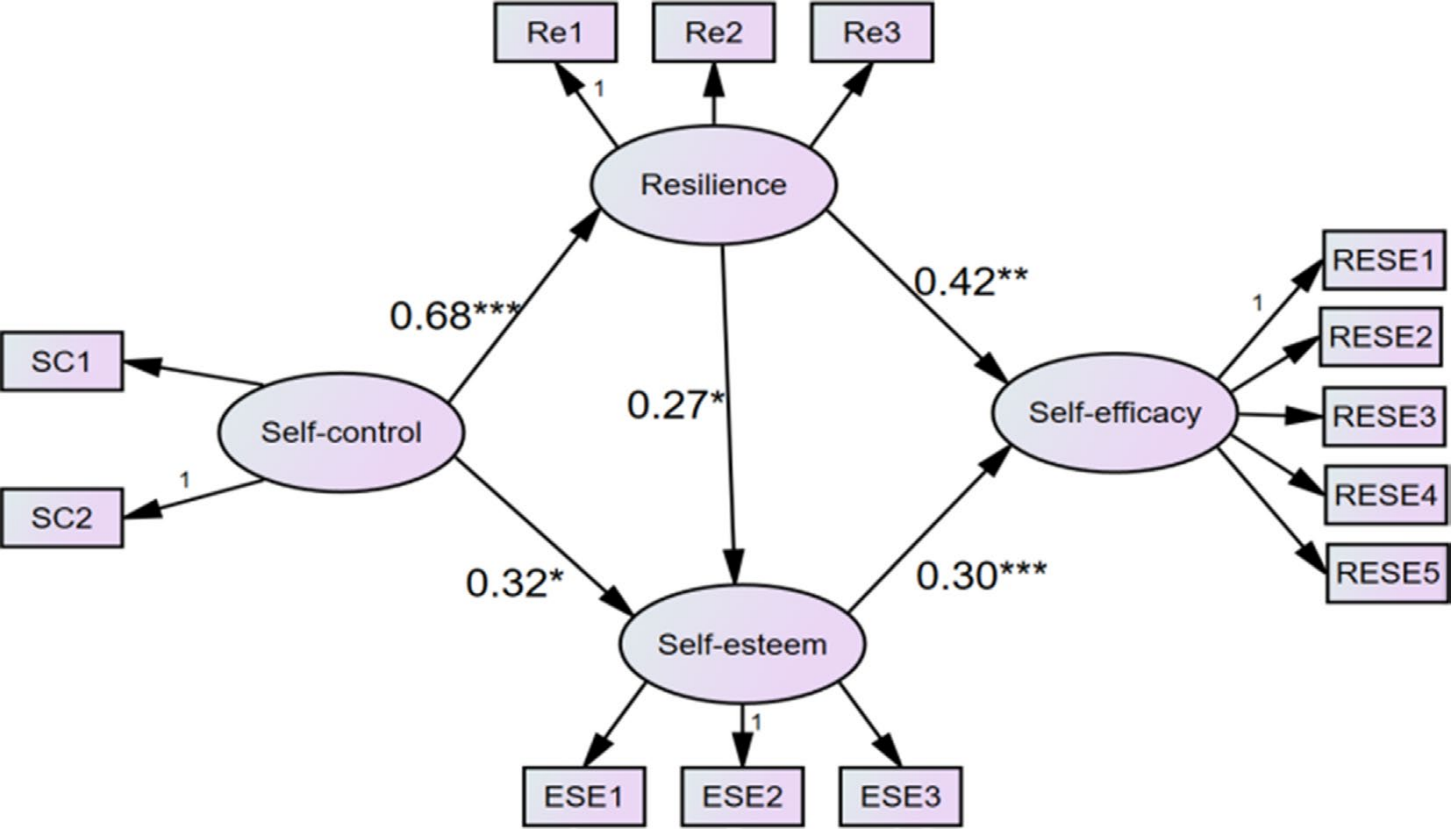
The finalized structural model (N = 298) in the present study. Note. Factor loading is standardized. SC1-SC2 = Two parcels of self-control; Re1-Re3 = Three parcels of Resilience; ESE1-ESE3 = Three parcels of Self-esteem. RESE1-RESE5 = Five parcels of regulatory emotional self-efficacy.

To further explain the mediating model, we built Model 4 that reversed the paths among self-efficacy, self-esteem, and resilience by controlling self-control (i.e., from self-efficacy to self-control and resilience followed by self-esteem to resilience), to test an alternative causal hypothesis. Although nearly all indices in Model 4 were inconsistent with the data, the GFI and CFI values were greater than 0.900 [(NCχ^2^/df) = 3.565, RMSEA = 0.093, SRMR = 0.1067, GFI = 0.908, TLI = 0.872, CFI = 0.909, and AIC = 268.054], and the standardized path coefficients between self-esteem and resilience were not significant in Model 4. In addition, the indices of AIC and ECVI in Model 3 were smaller than those from Model 4, so Model 3 was chosen as the most suitable model for evaluating the mediating effects (**Table 3**). The final structure model is shown in **Figure 2**.

**Table 3:** Fit indices among competing models.

Regression weights	Model1	Model2	Model3	Model4	Target value
Self-control→Self-efficacy		0.142			
Self-control→Self-esteem	0.558***	0.546***	0.315*	0.352***	
Self-esteem→Self-efficacy	0.304**	0.250*	0.303**		
Self-control→Resilience	0.719***	0.715***	0.679***	0.525***	
Resilience→Self-efficacy	0.434***	0.352**	0.421***		
Self-esteem→Resilience				0.103	
Resilience→Self-esteem			0.190*		
Self-efficacy→Resilience				0.366***	
Self-efficacy→Self-esteem				0.384***	
χ^2^	154.070	152.882	**149.968**	196.054	
df	56	55	**55**	55	
χ^2^/df	2.751	2.780	**2.727**	3.565	
SRMR	0.0561	0.0563	**0.0539**	0.1067	<0.06
RMSEA	0.077	0.077	**0.076**	0.093	<0.08
GFI	0.924	0.925	**0.926**	0.908	>0.90
TLI	0.912	0.911	**0.914**	0.872	>0.90
CFI	0.937	0.937	**0.939**	0.909	>0.90
AIC	250.070	250.882	**221.968**	268.054	
ECVI	0.842	0.845	**0.747**	0.903	

*Correlation is significant at the 0.05 level (two-tailed).

**Correlation is significant at the 0.01 level (two-tailed).

***Correlation is significant at the 0.001 level (two-tailed).

We used the bootstrapping procedures method of AMOS24.0 to test the significance of the mediated models. Based on recommendations of MacKinnon et al. (68), we generated 10,000 samples by random sampling of the original dataset (N = 426). If the 95% confidence interval for the outcome of the mediation effect did not contain zero, the mediation effect would be significant at the 0.05 level, and **Table 4** shows the indirect effects and their associated 95% confidence intervals, revealing that self-esteem and resilience exerted significant indirect effects on self-control and self-efficacy.

**Table 4:** Bootstrapping indirect effects and 95% confidence intervals (CI) for the final mediational model.

Number	Model pathways	Point estimates β	95%CI	
			Lower	Upper
1	Self-control→Resilience→Self-efficacy	0.1046	0.0346	0.1902
2	Self-control→Self-esteem→Self-efficacy	0.0516	0.0146	0.1050
3	Self-control→Resilience→Self-esteem→Self-efficacy	0.0043	0.0009	0.0132
4	Self-control→Resilience→ Self-esteem	0.0920	0.0546	0.1294
5	Resilience→ self-esteem→Self-efficacy	0.0181	0.0061	0.0389

### Gender Differences

There was no statistically significant gender difference in terms of self-efficacy, resilience, and self-esteem, although females scored higher than males with respect to self-control.

To further examine gender differences in the intermediary model, we conducted a multigroup analysis to explore whether the path coefficients differed significantly for males and females. According to Byrne (69), we compared gender difference using the following two models: 1) an unconstrained model, allowing all the paths to vary across both male and female groups; and 2) a constrained model, constraining all the parameters, including factor loading, error variances, and structure covariance, to be equal across male and female groups. After confirming the moderating effect of gender, we examined mediating models for males and females separately, with results indicating that differences between these two models were not significant [Δχ2 (18) = 26.480, p = 0.089]. Furthermore, in accordance with Arbuckle (70), we used the critical ratios of differences (CRDs) to judge the difference between two parameter estimates, measured by dividing the difference between two estimates by an estimate of the standard error of the difference. If a CRD value is greater than 1.96 (or 2.58), the two parameters were estimated to be significantly different at levels of p < 0.05 (or p < 0.01). Since the CRD analysis in this study indicated no structure path identified as significantly different, the finding indicated that the relationship among self-control, resilience, self-esteem, and self-efficiency did not differ with respect to gender.

## Discussion

Following the methodology of previous literature, in this study, we analyzed the relationship between self-control and self-efficacy among patients with substance use disorder by investigating the mediating role of resilience and self-esteem with respect to the impact of self-control on self-efficacy. The results indicated that self-control is positively related to resilience, self-esteem, and self-efficacy, indicating that our hypothesis is correct.

The mediating impacts of resilience and self-esteem with respect to the relationship between self-control and self-efficacy were identified as significant, with results indicating that participants exhibiting a more positive performance on the self-control scale performed better in terms of resilience and self-efficacy compared with participants exhibiting poorer self-control performance. These results are consistent with those of previous studies that also found resilience to be positively associated with self-control (36, 71) and self-efficacy (43, 72). Furthermore, participants who performed better in self-control were also identified as achieving more positive performance with respect to self-esteem and self-efficacy, in accordance with previous studies that found self-esteem to be closely correlated with self-control (22, 45) and self-efficacy (48, 49).

The combined theoretical underpinnings of this study might inform some practical implementations regarding patients’ substance use disorders. The theoretical underpinnings of intermediary mechanisms between self-control and self-efficacy reflect the idea that success in resisting inappropriate temptations (self-control) might contribute to developing propensity toward enduring pressure or negative emotions (resilience) and achieving greater self-confidence (self-esteem) in ways that enhance chances for individual success in overcoming challenging and resource-demanding tasks (self-efficacy). This suggests that there might be types of practical interventions for enhancing self-efficacy in patients with substance use disorders. For example, interventions might pay closer attention to designing specialized and supervised trait-enhancement programs in ways that provide evidence-based events focusing on fostering traits of self-control, resilience, and self-esteem. Overall, the present findings provide evidence of a psychological process whereby self-control exerts benefits on drug dependents to promote their self-efficacy *via* improved resilience and self-esteem.

Using the final model of this study, we found that the path “self-control →resilience →self-esteem → self-efficacy” was significant and showed that individuals with higher self-control are prone to experience higher levels of resilience, possibly enhancing their self-esteem and, in turn, producing a greater sense of self-efficacy. For one thing, this path suggests that resilience is a mediator between self-control and self-esteem, agreeing with earlier studies that resilience is significantly associated with self-control (40) and self-esteem (73). This path also demonstrates that self-esteem might act as a mediator between resilience and self-efficacy, an idea consistent with findings that resilience can play a crucial role in promoting self-esteem (74), and self-esteem is closely correlated with self-efficacy (49). Based on these findings, it is reasonable to speculate that resilience might play a mediating role in the relationship between self-control and self-esteem, while self-esteem might act as a mediator between resilience and self-efficacy.

The results of this study also indicated that females experience higher levels of self-control than males. The results related to self-control were in agreement with previous studies suggesting that females tend to regulate themselves better than males (75), probably because the female’s prefrontal cortex that dominates the functions of self-control is more active than that of the male (75). However, the final model did not suggest gender differences, rather indicating that both males and females have the same mediating mechanisms between self-control and self-esteem.

In short, the study broadens our horizon with respect to the complicated interplay between self-control, resilience, self-esteem, and self-efficacy among patients with substance use disorder in China. Considering the significant path from self-control through resilience and self-esteem to self-efficacy sheds light on potential mechanisms linking self-control and self-efficacy. This study may also offer valuable evidence on how to organize psychological interventions that aim to promote self-efficacy of patients with substance use disorders. Encouraging self-control, resilience, and self-esteem in the future would work as proactive tools, helping them enhance self-efficacy.

## Limitations

This study, like most studies, has some limitations, the main one being the lack of a control group. Also, all the information was collected by questionnaires and scales that can be influenced by subjectivity. Second, to moderate such adverse impacts, it is also recommended that multiple assessment methods such as structured interviews be introduced (SCD-1) to support more in-depth and accurate diagnoses. Third, the diagnostic questionnaire did not cover assessment of histories of comorbid disorders and psychotropic mediation that have been identified as significant variables by many studies of substance use disorders (76, 77). Furthermore, the results were based only on two-dimensional measures of resilience and self-esteem, and in future studies, it might be useful to examine other facets of self-control and explore effects of other possible mediating factors such as social support, affect, and loneliness, on the relationship between self-control and self-efficacy. Finally, the study’s sample population’s age range was from 18 to 64, so it remains to be seen whether the results could be duplicated with younger or older participants.

## Ethics Statement

This study was approved by the ethics committee of Nanjing Medical University. All participants volunteered to participate in the study and signed informed consent documents.

## Author Contributions

CY, YZ, QC, MX and JA conceived this work. CY conducted the development of the manuscript and contributed to the modeling and data analysis. YZ contributed literature review, discussion, revision and polish. QC, MX and JA revised and finalized the manuscript. All authors read and approved the final manuscript.

## Funding

This paper was supported by the Research Fund for Philosophy and Social Science of Universities in Jiangsu Province (2017SJB1800), China Postdoctoral Science Foundation (2018M632285), and Jiangsu Province Social Science Fund Project (18SHC006).

## Conflict of Interest Statement

The authors declare that the research was conducted in the absence of any commercial or financial relationships that could be construed as a potential conflict of interest.
